# Feasibility and acceptability of a contextualized brief psychological intervention for people with bipolar disorder in rural Ethiopia

**DOI:** 10.1186/s40814-025-01683-9

**Published:** 2025-07-17

**Authors:** Mekdes Demissie, Charlotte Hanlon, Lauren C. Ng, Rosie Mayston, Abebaw Fekadu

**Affiliations:** 1 Department of Psychiatry, College of Health and Medical Sciences, Haramaya University, Dire Dawa, Ethiopia; 2 Centre for Innovative Drug Development and Therapeutic Trials for Africa (CDT-Africa), Addis Ababa University, Addis Ababa, Ethiopia; 3 Division of Psychiatry, Centre for Clinical Brain Sciences, The University of Edinburgh, Edinburgh, UK; 4 Department of Psychology, University of California, Los Angeles, Los Angeles, USA; 5 Global health and social medicine, King’s College London, NE Wing Bush House, 30 Aldwych, London, WC2B 4BJ UK; 6 Department of Psychiatry, College of Health Sciences, Addis Ababa University, Addis Ababa, Ethiopia; 7 Department of Psychological Medicine, Center for Affective Disorders, Institute of Psychiatry, Psychology and Neuroscience, King’s College London, London, UK; 8 Department of Global Health & Infection, Brighton and Sussex Medical School, Brighton, UK

**Keywords:** Fidelity, Intervention development, Primary health care, Intervention integrity, Low- and middle-income countries

## Abstract

**Background:**

There is a very large unmet need for appropriate psychological interventions for bipolar disorder (BD) for use in low- and middle-income countries. We developed a psychological intervention for BD in a primary health care (PHC) setting in Ethiopia using the Medical Research Council’s framework for the Development and Evaluation of Complex Interventions. The aim of this study is to investigate the feasibility, acceptability, and fidelity of this newly developed psychological intervention for BD in a PHC setting in south-central Ethiopia.

**Method:**

A total of 12 euthymic people with bipolar disorder and five caregivers participated in five 20-min weekly sessions of the psychological intervention. We conducted a mixed-method evaluation, including in-depth qualitative interviews, fidelity ratings of a random selection of 25% of the audio recorded intervention sessions, and self-reported change in symptom severity. We used thematic analysis for qualitative data and descriptive analysis for quantitative data.

**Results:**

Except for one caregiver, all participants completed all five sessions. Intervention providers and recipients expressed satisfaction with the intervention. Intervention providers reported that the intervention can be feasibly delivered in the PHC setting, although 20 min was considered insufficient. While participants acknowledged the importance of involving caregivers in the intervention, they raised privacy concerns. Intervention providers’ adherence to the manual was moderate. Preliminary findings indicate a reduction in depressive symptoms post-intervention and improvement in providers’ perceived knowledge and skills.

**Conclusions:**

This contextually developed psychological intervention for bipolar disorder has promising feasibility, acceptability, and potential utility. Further studies should evaluate time considerations and effectiveness.

**Trial registration:**

The trial was registered on 16 August 2024, retrospectively on the Pan African Clinical Trial Registry database [PACTR202408896160144], https://pactr.samrc.ac.za/TrialDisplay.aspx?TrialID=31727.

**Supplementary Information:**

The online version contains supplementary material available at 10.1186/s40814-025-01683-9.

## Key messages regarding feasibility

- *What uncertainties existed regarding the feasibility?*

We wanted to understand the feasibility, acceptability, and potential benefits of delivering psychological interventions by non-specialists in primary care to people with bipolar disorder (BD) and their caregivers.

- *What are the key findings?*

Non-specialists demonstrated adherence and competency in delivering a psychological intervention to people with BD and their caregivers. The intervention was rated as feasible and acceptable by providers and participants. Providers also emphasized the usefulness of training resources, such as local-language manuals and role plays.

- *What are the implications of the findings for the design of the main study?*

To enhance the intervention’s effectiveness, extending session duration, providing ongoing support for non-specialists, and continuing caregiver involvement are crucial. The intervention should allow flexibility to tailor sessions to participant needs while preserving core components. Future studies need to include strong measures to evaluate clinical outcomes, provider skills, and participant satisfaction for comprehensive improvement.

### Background

Bipolar disorder (BD) is a disabling condition. In 2017, the global disability-adjusted life years (DALYs) for BD reached 9.29 million, an increase of 54.4% since 1990 [1]. Studies from low- and middle-income countries (LMICs) indicated that BD is associated with a high relapse rate [2], suicidality [3], stigma [4], premature mortality [5], and functional impairment [6]. Psychological interventions reduce symptom burden and improve functioning in people with BD [7, 8]. The use of culturally adapted or developed psychological interventions increases acceptability and feasibility [9, 10]. Various studies have reported that cultural adaptability [11], treatment fidelity [12], and competency of the intervention providers [13] are associated with the effectiveness of psychological interventions. Moreover, acceptability of the intervention to both intervention providers and recipients affects the successful implementation of the intervention [14]. The lack of acceptability of interventions reduces adherence and its effectiveness [12]. To address the unmet needs and priorities of people with BD in Ethiopia, we developed a contextually appropriate, brief, psychological intervention [15] using the Medical Research Council’s (MRC) Framework for the Development and Evaluation of Complex Interventions [16]. The aim of this study was to investigate the feasibility, acceptability, and fidelity of this psychological intervention for people with BD in routine primary health care (PHC) clinic settings, and to understand the feasibility and acceptability of providing the intervention with or without their caregivers’ presence.

## Methods

### Study setting

This study was conducted in the Butajira and Sodo districts of the Gurage Zone, Southern Nations, Nationalities, and Peoples region (SNNPR), Ethiopia in June to August 2021. The study was carried out in primary health care centers. Sodo district has eight health centers and one primary hospital, whereas Butajira and its surroundings have one district hospital and 13 health centers [17]. In this feasibility study, we included two health centers, one primary hospital, and one district hospital. Details of the study site are described elsewhere [18, 19].

### Brief description of the intervention

We used the Medical Research Council’s (MRC) framework for the Development and Evaluation of Complex Interventions integrated with a Theory of Change (ToC) approach to develop a manualized psychological intervention for people with bipolar disorder in rural Ethiopia [15, 20]. The MRC framework recommends four phases to develop and evaluate complex interventions: intervention development, feasibility and piloting, evaluation, and implementation [16]. During the intervention development phase, we reviewed evidence for effective psychological interventions for people with BD in LMICs [8], conducted a qualitative study to understand the unmet needs and priorities of people with BD in Ethiopia [18], and convened ToC workshops with key stakeholders to develop contextually relevant psychological intervention components [15]. In this paper, we evaluated the feasibility and acceptability of the intervention (the second phase of the MRC framework).

The developed psychological intervention [15] has five sessions which are (i) initial engagement which focuses on needs assessment and goal setting; (ii) psychoeducation about bipolar disorder symptoms, early warning signs of relapse, and the causes and influencing factors for bipolar disorder; (iii) treatment and treatment adherence including treatment options, medication side effects, and treatment adherence; (iv) promoting wellness through problem-solving techniques and sleep hygiene; and (v) anxiety management and relapse prevention planning through use of muscle relaxation and breathing exercise, and preparing and implementing an action plan to prevent relapse. In addition to the manual, patient and provider information leaflets were provided to facilitate the intervention sessions. The intervention manual and leaflets were developed in the local language Amharic with the help of service users, caregivers, and local health providers and community members.

Each session was intended to last for 20 min and to be delivered by PHC providers, once a week, for 5 weeks. The intervention was delivered in the health facility where the person with BD attended for their regular follow-up care. Session timings were arranged in consultation with the participants. A total of six people with BD attended the sessions alone, while another six participated in the intervention with their caregivers.

#### Intervention providers

Four Bachelor of Science (Bsc.) level PHC providers (three men and one woman) and two male supervisors participated in the feasibility trial. The eligibility criteria for the providers were (1) being able to attend all the training sessions, (2) expressing interest in taking part in the study, and (3) being trained on the World Health Organization Mental Health Gap Action Programme intervention guide (mhGAP-IG). All intervention providers had been treating people with mental illness in the PHC setting for at least 3 years.

#### Provider training

Providers received 2 weeks of training in the PSI-BE, including 1 week of didactic training and 1 week of role play delivering the intervention with supervision by the trainers. The training was facilitated by the first author (MD) who led the intervention development, and a clinical psychologist. Both facilitators have Master of Science (MSc) degrees and previous experience delivering psychological interventions to people with mental illness, and are familiar with the study setting.

#### Participant recruitment

Participants were selected purposively based on their gender, age, place of residence, socio-economic status, and clinical status to test the feasibility of the intervention with patients from diverse backgrounds. First, mhGAP-IG trained PHC providers who were treating people with mental illness at outpatient clinics used medical records to identify people with BD who had regular psychiatric follow-up care. The mhGAP-trained PHC workers list only the diagnosis and are not expected to specify the type of bipolar disorder (Type I or Type II) in the PHC settings. Additionally, we did not hypothesize a difference in the feasibility or acceptability of the intervention for patients with type I vs type II BD. Therefore, in this study, we included any individuals with BD regardless of the type. Second, psychiatric nurses assessed whether the patients were eligible based on their clinical judgment (see Table 1 for inclusion criteria). Third, research field workers approached the potential participants at their homes, informed them about the study, and invited them to participate. Those who provided informed consent were linked to the intervention providers.

**Table 1:** Eligibility criteria for study participants

Participants	Eligibility criteria
People with BD	✓ Age 18 or above ✓ Diagnosed with BD and engaged in treatment during the study period ✓ Willing to attend five consecutive weekly sessions ✓ Determined by psychiatric nurse clinical assessment to be euthymic and able to give informed consent and participate in the study
Caregivers	✓ Age 18 or above ✓ Immediate caregiver of a patient with BD ✓ Willing to attend five consecutive weekly sessions.

### Sample size

As this is a feasibility study aimed at assessing the feasibility and acceptability of implementing the intervention, we did not conduct a formal sample size calculation. Instead, we included a total of 18 participants (12 people with bipolar disorder and 6 caregivers), based on the available resources (time and budget). This aligns with the recommended minimum sample size of approximately 12 participants for pilot or feasibility studies [21]. Additionally, most of the outcomes were assessed qualitatively. Of the 12 individuals with bipolar disorder, six participated in the intervention alone, while the remaining six participated alongside their primary caregiver, who provides support to them.

### Outcome and measures

We used a mixed-methods approach to investigate the feasibility, acceptability, fidelity, change in patients’ BD symptom severity, and perceived utility of the intervention. See Table 2 for study outcomes and measurement.

**Table 2:** Primary and secondary outcomes

Outcomes		Measures
Primary outcome	Feasibility	Number and percent of people with BD who: ▪ Were approached and were willing to participate ▪ Dropped out before finishing the intervention ▪ Completed the intervention
	Acceptability	Qualitative reports of satisfaction with the intervention by providers, patients, and caregivers
	Adherence to the intervention manual and competency	Clinical psychologists rated 25% of the recorded intervention sessions using adherence to the manual measure developed for this project
Secondary outcome	Change in knowledge and skill of intervention providers	Pre- and post-training assessment of perceived knowledge and skills
	Change in symptom severity	Before and after intervention assessment using Patient Health Questionnaire-9 and Young Mania Rating Scale for mania symptoms

#### Feasibility of intervention

We recorded the number of people with BD and their caregivers who were approached and agreed to participate. We also recorded the number of sessions completed by participants.

#### Acceptability of intervention

We conducted semi-structured interviews with people with BD, caregivers, and intervention providers to explore satisfaction with the intervention, understandability of the content, challenges experienced during the intervention process, and perceptions of the benefits or harms of the interventions. All interviews were conducted in Amharic by two experienced qualitative researchers who were not involved in training or delivery of the intervention. Participants were interviewed in a private room, either in the facilities where they received the intervention or in the project office, based on their preference. The interviews were conducted 1 week after completion of the intervention. Interviews lasted from 20 to 40 min, and all were audio recorded.

#### Adherence and competency

We assessed the competency and adherence to the manual (i.e., fidelity) by rating the recorded intervention sessions. It is recommended that fidelity can be assessed by comparing the content in 20 to 40% of recorded intervention sessions to a prespecified criterion [22, 23]. Thus, all intervention sessions were audio-recorded, which resulted in a total of 60 audio records from 12 participants. A fidelity checklist was developed based on the manual and piloted prior to the start of the intervention study. The checklist consisted of two sections (Supplementary material 1): (i) the first section included four items that assessed the competency of the intervention providers, such as using appropriate language, starting with open-ended questions, being sensitive to participants’ explanations, and effectively managing time to meet session goals; and (ii) the second section included items assessing components specific to the session.

Adherence to the manual was rated using a 5-point scale ranging from 1 (very poor) to 5 (very good). According to the manual, mean ratings score below 3 indicate poor adherence, a rating of 3 indicates moderate adherence, and ratings above 3 indicate high adherence [19]. Two master’s degree clinical psychologists, who were not part of the research team, conducted the ratings of the recorded intervention sessions. First, they randomly selected three recorded interventions from each session, which resulted in rating 25% (15/60) of the total records. Each rater then listened to the selected records independently and rated them using the checklist. Finally, for any discrepancies in ratings, the two raters listened to the records together and reconciled their ratings through discussion. Additionally, when they completed the ratings, they also documented their observations on the quality of the intervention delivery and areas that required further training or improvement.

#### Change in knowledge and skill

We used self-administered pre- and post-training assessment questionnaires to investigate changes in the knowledge and skills of intervention providers. The questionnaires had eight knowledge-related items and eight items linked to skills. Each item was rated on a Likert scale that ranged from 1 (very poor) to 5 (excellent). The items focused on the following areas: (a) symptoms and causes of BD (3 items), (b) treatment (3 items), (c) promoting wellness and managing anxiety (1 item), and (d) core skills in the psychological intervention (1 item). The total score ranged from 3 to 15 for the domains with 3 items and from 1 to 5 for those with 1 item.

#### Change in symptom severity

The Young Mania Rating Scale (YMRS) [24] and Patient Health Questionnaire-9 (PHQ-9) [25] were used to assess the severity of manic and depressive symptoms, respectively. PHQ-9 has 9 items with item scores ranging from 0 to 3, and the total score ranges from 0 to 27. Whereas, YMRS has 11 items with item scores ranging from 0 to 4, and the total score ranges from 0 to 44. Both assessment tools were used as continuous variables, with the higher score indicating greater severity of symptoms [24, 25]. Both instruments were previously translated and used in Ethiopia [26, 27]. In the PHC setting, mhGAP-trained workers were only expected to record a diagnosis of bipolar disorder without specifying the subtype (Type I or Type II). The questionnaires were administered by PHC workers who had been trained in the mhGAP intervention guide but were not involved in the direct provision of the intervention.

### Analysis

For the qualitative data, we used the thematic analysis approach [28]. First, interviews were transcribed verbatim, then translated to English by experienced and independent translators. Then, the transcripts were imported into Open Code 4.03 [29]. The first author (MD) coded two randomly selected transcripts and developed a codebook, and then discussed the codes and codebooks with the second author (CH). Then, MD coded the remaining transcripts based on the codebook. In the second stage, we grouped similar or related codes into clusters to capture the essence of particular themes.

For the fidelity assessment, the consensus scores obtained from the two raters for each item within a session were averaged to get the mean score for the session. We obtained the overall fidelity measure across all the sessions by calculating the mean score of all items across the five sessions (*n* = 15). We used descriptive statistics with minimum, maximum, and median scores to calculate changes in symptom severity of people with BD and intervention providers’ knowledge.

## Results

### Socio-demographic characteristics

All twelve people with BD and the six enrolled caregivers in the condition that included BD–caregiver pairs who were invited to participate agreed (100% agreement rate). Most of the people with BD had formal education, and half of them were married. Additionally, most had been living with BD for longer than 3 years, and most experienced at least one relapse after being diagnosed with BD. See Table 3 for complete demographics.

**Table 3:** Socio-demographic and clinical characteristics of the study participants

	Socio-demographic variables		Number
People with bipolar disorder	Age in years	Mean (SD)	32.6 (11.1)
	Sex	Female	7
		Male	5
	Educational status	Non-literate	2
		Primary	5
		Secondary or tertiary	5
	Marital status	Single	6
		Married	6
	Number of relapses since the onset	No relapse	2
		1–2 relapse	4
		3–5 relapse	4
		> 5 times	2
	Duration of illness	< 2 years	3
		2–5 years	4
		> 5 years	5
Caregivers	Age in year	Mean (SD)	41.2 (8.7)
	Sex	Female	2
		Male	3

### Feasibility of the intervention

All people with BD [12] and six caregivers completed the first session and all, except one caregiver, completed all five sessions. The caregiver dropped out after the second session because of a scheduling conflict with his new job.

#### Feasibility of intervention duration

Based on the recorded intervention sessions, the average duration of the sessions was 33 min (sd = 5.1), which ranged from a mean of 25 min to complete session 1, to 39 min to complete session 4. During the qualitative interviews, intervention providers mentioned that the sessions took longer than the expected 20 min due to participants’ need to know more about their condition, discuss social issues, and review the previous session before beginning the day’s session. Additionally, they specifically mentioned that some sessions required more time to practice new skills with participants.

“Some patients ask more questions and need to discuss more…. Session three and session four took me about 40 minutes to deliver because these sessions need time to practice. Hence, the time allocated needs to be revised” (Intervention provider, ID #02).

Another intervention provider mentioned that patients want to share their life experiences during the intervention, and interrupting them could discourage them and also affect their relationship with the participants.

“Patients want to share their personal experiences, and they want to be listened to. Sometimes, they might cry when they recall their previous experiences. Thus, discussing those issues takes time, and it is not always possible to do all of that within 20 minutes.” (Intervention provider, ID #01).

People with BD and caregivers mentioned that even though the duration was longer, they did not feel worried or bored because sessions were provided at convenient times, which also contributed to their high attendance.

“I was attending the sessions after I had finished all the household chores. I also informed my family that I would have an intervention and got permission from them. This was about 30 or 40 minutes, but I was not worried about the work I had when I got back home. So, I’m okay with the time.” (Person with Bipolar Disorder, ID #01)

Professionals who rated the recorded intervention sessions reported that intervention providers spent quite a bit of time reviewing the previous sessions before starting the day’s session, contributing to the longer duration of intervention. The intervention providers suggested making all sessions 30 min and making the total number of sessions 6 rather than 5.

#### Acceptability of the intervention content and format

Participants with BD and their caregivers mentioned that they were ready to participate in the intervention due to the perceived benefit. One caregiver spoke of his motivation:

“Of course, if people are not convinced, sitting for 10 minutes could be difficult. But, if they understand that the treatment is for their own benefit, an hour could be tolerable. You have given us this education to improve the health of my wife. So, how could I feel tired to learn?”

Participants also mentioned that they found the intervention useful and supported their coping efforts, though the session perceived to be the most important differed depending on their priority problems. Some participants said that education about illness and treatment was most helpful because it had helped them to improve their knowledge about their own or their relative’s illness and treatment. One person with BD said:

*“*I have learned a lot about my illness and the treatment. I learned that the medication will help me to feel calm and have a good relationship with my family. I have also learned why I need to take medication for a long time and the negative effect of stopping it on my health” (Person with Bipolar Disorder, ID #12).

Caregivers also acknowledged the usefulness of the session that described medication treatment, reporting their satisfaction as follows:

“In general, the session on medication was most important. It (medication) is very helpful for her (patient)to stay well; it helps her to live a normal life with the family, neighbors, and with the community. It is also important for us as a family because if one person gets unwell in the household, the whole family gets affected.*”* (Caregiver, ID #02).

Other participants mentioned the content on “how to improve sleep” as most important. They mentioned that sleep problems are one of the major challenges for people with BD. People with BD described the importance of this session as follows:

“I am happy because I have learned how to improve my sleep. Now, I know the importance of sleeping at a regular time and waking up at the same time. Now, I am trying to bring that practice. I also stopped drinking coffee at night*.”* (Person with Bipolar Disorder, ID #08).

Regarding anxiety management techniques (muscle relaxation and breathing exercises), participants reported different experiences. Some found it hard and needed more practice to master the exercises; others found the exercises easy to practice, and they liked it best of all aspects of the intervention.

“I liked the exercise session most because they teach us how to reduce our tension by using it. The inhaling and exhaling part of the exercise is very enjoyable to me and not very difficult” (Person with Bipolar Disorder, ID #04).

Intervention providers also reported that participants were happy during the intervention sessions, even though they noticed different levels of understanding among participants. As a result, the same topic took a different amount of time to cover for different people with BD and caregivers. One intervention provider explained the situation as follows:

“Participants were happy during the intervention sessions but, they have a different level of understanding. They understand most of the intervention session but not all…especially illiterate patients need more time.” (Intervention provider, ID #02).

Regarding the intervention format**,** both people with BD and their caregivers who received the intervention together highlighted the importance of engaging caregivers in the intervention. When caregivers were involved, people with BD were pleased with their involvement. The reason was that caregivers learned more about their relative’s illness and started to understand the person better than before they received the intervention.

“My husband started to understand my illness after he took the education. Now, he even tells the children not to disturb me; he advises me to finish the chores and sleep early. Now, he is starting to understand me because he has learned about my illness” (Person with Bipolar Disorder, ID #01)

Intervention providers, on the other hand, identified the difficulties of discussing family-related issues with people with BD in the presence of their caregiver or vice-versa, despite believing that involving caregivers in the intervention was important because of the essential role that caregivers play in the lives of people with BD.

“Sometimes, there are family-related issues like disagreement or other family-related issues that the patients perceived as a cause or triggering factor for their illness. Thus, they feel discomfort talking about it in the presence of their family, and they need to discuss it alone. So, it is good to consider both sides.” (Intervention provider, ID #03).

#### Perceptions of intervention providers on training and the manual

Intervention providers liked the way that the training material had been prepared, including the color printing and the instructions given to intervention providers. They also mentioned that preparation of a manual in the local language helped them to understand better. All of the intervention providers mentioned that the content covered by the manual was sufficient to help them deliver the intervention. They also specifically mentioned that the communication skills component was most useful because of its applicability for any patients, whether they had a physical or mental illness.

“Communication skills were one of the sessions that received a lot of attention during the training. Since we are not mental health experts, we had a communication gap. Previously, we were relying more on medicine than psychological intervention. This section is useful to build good communication with other patients as well.” (Intervention provider, ID #01).

Regarding the training approach, professionals appreciated the use of case stories, role plays, and experience sharing.

“During the training, we were discussing a hypothetical case based on our previous experience. For example, we were saying what if the participant possibly asks this and that question and how we can provide an answer for them and the like… which really helped us to understand the topic’ (Intervention provider, ID #03).

Another intervention provider also mentioned the importance of communication skills as follows.

“We have discussed how to create trust, which is a great idea. We do not always inquire about issues that are not clearly described by the patient. For example, we do not usually check if patients have had a history of suicidal attempts or ideation. Therefore, the skill we got from the current training was important.” (Intervention provider, ID #02).

Finally, the intervention providers described that the providers’ information leaflet was helpful, especially to quickly review the content of the intervention during intervention provision. However, the font size was considered to be too small and the double-sided printing affected how it could be used.

### Findings of pre-post knowledge and skill assessment from intervention providers’ perspective

As shown in Table 4, the study presents the results of the pre- and post-training assessments from the intervention providers. For the domains with three items, the total theoretical range of scores is from 3 to 15, while for the one-item domains, the range is from 1 to 5. Providers demonstrated improvements in both knowledge and perceived skills related to BD following the training. Specifically, intervention providers demonstrated a better understanding of BD symptoms, treatments, wellness techniques, and core psychoeducation skills, with mean scores and medians increasing across all areas in the post-training assessment. In the post-training assessment, providers’ perceived skills in teaching BD symptoms and treatments, as well as in demonstrating wellness techniques, also showed improvement compared to the pre-training evaluation. Overall, the training proved to be effective in enhancing providers’ knowledge of BD and boosting their confidence in teaching and applying relevant skills, as reflected in the overall improvement in scores.

**Table 4:** Change in providers’ knowledge and self-reported skills (*n *= 9)

Items used as a measurement		Mean (SD)		Median (IQR)	
		Pre-intervention	Post-intervention	Pre-intervention	Post-intervention
Knowledge	Knowledge on symptoms (3 items)	11(1.5)	14.4 (1.1)	11 (10, 12)	15 (14, 15)
	Knowledge about treatments of BD (3 items)	12 (1.6)	13.8 (0.8)	12 (10.5, 13)	14 (13, 14.5)
	knowledge of techniques used to improve wellness (PST, sleep hygiene, anxiety management (1 item)	3.5 (0.7)	4.4 (0.5)	3 (3, 4)	4 (4, 5)
	knowledge on the core skills in psychoeducation and psychosocial intervention (1 item)	3.7 (0.7)	4.4 (0.5)	4 (3,4)	4 (4,5)
Perceived skill	Teaching the sign and symptoms of BD (3 items)	9.8 (2.2)	14.5 (1.0)	9 (9, 10.5)	15 (14,15)
	Teaching about the treatment of BD (3 items)	10.8 (1.6)	14.4 (0.9)	11 (9.5, 12)	15 (13.5, 15)
	Demonstrating the techniques used to improve wellness (1 item)	3.3 (0.9)	4.4 (0,5)	3 (3, 4)	4 (4,5)
	Using the core skills in psychoeducation and psychosocial intervention (1 item)	3.2 (0.9)	4.2 (0.4)	3 (2.5, 4)	4 (4, 4.5)

*IQR* =interquartile range, *BD* =Bipolar disorder, *SD* = standard deviation

### Fidelity of intervention delivery by intervention providers

**Fig. 1:**
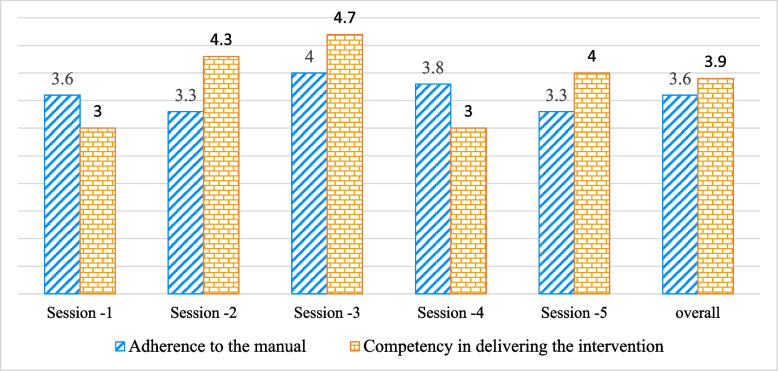
The adherence and competence of PHC workers in delivering the intervention

Figure 1 illustrates the adherence and competence of PHC workers in delivering the intervention. The average adherence of PHC workers to sessions ranged from 3.3 to 4. Sessions 2 (psychoeducation about the signs and symptoms of BD and early warning signs of relapse) and 5 (anxiety management and relapse prevention plan) had a lower mean score (3.3 out of 5), while the third session (psychoeducation about treatment) achieved the highest mean score (4 out of 5). According to the manual, mean rating scores below 3 indicate poor adherence, a rating of 3 indicates moderate adherence, and ratings above 3 indicate high/good adherence [19]. Therefore, across 15 session records, the overall mean adherence score to the manual was 3.6, which exceeds the threshold and indicates good adherence.

PHC workers’ competency in delivering the intervention sessions varied between 3 and 4.7. Sessions 1 and 4 had lower mean scores (3.3 out of 5), while the third session achieved the highest mean score (4.7 out of 5). Overall, across 15 session records, PHCs demonstrated a competency of 3.9, which is above the threshold.

## The impact of the intervention on symptom severity

The median severity scores of depressive and manic symptoms before and after delivering the intervention are summarized in Table 5. Based on descriptive data, there was a reduction in depressive symptoms post-intervention compared to the pre-intervention results. However, the reduction in mania symptoms score was not different from pre-intervention.

**Table 5:** Depressive and mania symptom severity score before and after intervention (*n *= 12)

Symptoms of interest	Instruments used to measure the outcomes	Pre-intervention Median (Min, Max)	Post-intervention Median (Min, Max)
Depressive symptom severity	PHQ-9	4 (0,9)	1.5 (0,6)
Manic symptom severity	YMRS	1.5 (0,5)	1.5 (0,4)

*Min =*minimum, *Max =*maximum, *PHQ-9 =*Patient Health Questionnaire,*YMRS* =Young Mania Rating Scale

## Discussion

To the best of our knowledge, this is the first study to assess the feasibility and acceptability of a psychological intervention for people with BD that was developed to be contextually appropriate for the primary care setting in a low-income country. The developed intervention was well-received by people with BD, caregivers, and providers and led to perceived benefits, including reductions in depression symptoms. In addition, the providers’ knowledge about bipolar disorder, its treatment, and techniques used to improve wellness and anxiety management improved after the training. A systematic review on the effectiveness of psychological interventions for BD in LMICs found that all the included studies were conducted in teaching or university hospitals, with interventions delivered by mental health specialists, including psychiatric nurses and psychologists with a degree or master’s level, psychiatrists, or psychiatric residents [8], whereas in our study the providers are non-specialist, which may indicate that the non-specialist delivered interventions have promising effects on depressive symptoms among people with BD [30].

All participants except one caregiver attended all sessions, which supports the feasibility of the intervention [14]. This finding is comparable to a pilot study conducted in Pakistan that reported a 100% attendance rate for 12 sessions of psychoeducation for people with BD [31] and is better than other studies that tested the feasibility of 16–20 sessions of cognitive behavioral therapy for bipolar disorder (23–40% drop-out) [32, 33]. The high attendance rate of the intervention in our study could be due to a lower number of sessions, the participatory development of the intervention, involving all stakeholders [34, 35], and the efforts made to ensure that it would fit into the local context [9, 10]. With psychological interventions, difficulty finding a convenient time for sessions is a common barrier to attendance [36]. In the current study, the time flexibility for intervention provision helped the participants attend all the sessions and reduced the risk of dropping out of the intervention.

The psychological intervention was used to enhance the understanding of study participants about the illness, to help them to acquire skills used to cope with challenges [37] and to maintain their wellbeing [38]. In this study, participants reported that they had acquired improved knowledge and skills related to BD. However, the degree of importance of each session was different for different participants, indicating a need for the providers to personalize the focus of the intervention. People with BD have various needs related to symptoms of illness, treatment, quality of life, and their family relationships, which require different approaches [39]. Likewise, caregivers and people with BD may also have different priorities [18].

Regarding the symptoms severity, no change was observed in YMRS scores from pre- to post-assessment, which may be attributed to the low baseline scores, as all participants were in a euthymic phase and met inclusion criteria requiring the capacity to provide informed consent.

In contrast, the depressive symptoms showed a reduction following the intervention, which is encouraging and consistent with findings from systematic reviews of psychosocial interventions for BD [8, 40]. However, this result should be interpreted with caution due to the small sample size and absence of a control group. The initial pre–post effect sizes warrant further investigation in larger, controlled trials.

In the current study, the overall mean level of adherence to the intervention content and providers’ competency in delivering the intervention was moderate. This result is lower than the previous feasibility studies conducted in high-income countries, which found high fidelity in a family-focused intervention for schizophrenia [41] and youth at risk of bipolar disorder [42]. However, the difference in adherence may be due to differences in qualifications and years of experience in providing psychological interventions. In our study, PHC workers delivered the intervention for the first time, while in the previous studies [41, 42], providers were master’s or doctoral-level psychologists. Additionally, almost all sessions in our study took longer to deliver than the proposed 20 min. Intervention providers reported that the content was important and should not be reduced, instead suggesting that the last two sessions could be made into three sessions, and that 30 min was a more realistic timeframe for each session. A systematic review that synthesized published results in LMICs reported 3 to 12 individual sessions and the duration of each session ranged from 45 to 60 min [8] which is much higher than that allocated for each session in the current study. Therefore, taking the feasibility of time into account is needed during manual revision.

The findings of a previous systematic review indicated that family-focused psychological interventions were effective in reducing relapse and hospital admission [8]. During the intervention development, the Theory of Change (ToC) participants and mental health experts strongly suggested that caregivers be included in the treatment as long as this accorded with the preference of the person with BD [15]. During the feasibility study, however, intervention providers observed incidences of hesitation to freely discuss family-related issues both from people with BD and caregivers. Thus, understanding confidentiality issues and how family conflicts can be managed needs to be considered [43]. In group therapy, having one session with each member of the group before the actual group therapy is recommended as an important skill to understand the needs of each participant [44]. In the current feasibility study, intervention providers also suggested that there should be flexibility to allow providers to meet with the person with BD and caregiver individually whenever they find it to be necessary.

The study identified both strengths and challenges that are likely to occur during the actual implementation of the intervention. One strength of this study is that the intervention was based on the recommendations of the MRC framework, which we used to develop the intervention. Additionally, the involvement of various stakeholders during the intervention development was another strength of this study. The intervention was piloted in a routine clinical setting where the intervention was planned to be delivered. Additionally, we assessed the feasibility and acceptability of the intervention using multiple methods, including qualitative interviews and pre-post assessments, and fidelity was assessed using ratings of randomly selected intervention sessions. Moreover, we assessed the feasibility and acceptability of the intervention from service users, caregivers, and intervention providers’ perspectives. Third, the study identified areas that need further modification before testing effectiveness and will be used to refine the intervention. However, findings should be interpreted in the light of the following limitations: (i) the sample size was small, especially for caregivers, (ii) there was no comparison group due to the small sample size and limited resources, (iii) we did not quantify the change in knowledge and skills of intervention participants, (iv) the assessment of competency from the recorded intervention sessions may not capture nonverbal communication that is not amenable to audio recording, and (v) another limitation is that pre-specified criteria for deciding whether to proceed to the next stage were not considered during the study design. As a result, decisions about progressing to a full-scale trial were based on post-hoc interpretations of the data. To improve the transparency and rigor of feasibility assessments, future studies should define clear progression criteria in advance, aligned with the study’s objectives.

## Conclusion

The intervention evaluation approach focused on the feasibility and acceptability of the developed intervention in terms of the content, training manual and leaflet preparation, language, and delivery strategies that helped to improve stakeholders’ buy-in [45] and ensured scalability of the developed psychological intervention. The findings of this study suggest that the psychological intervention is feasible and acceptable to deliver by PHC workers in LMIC settings. Efficacy and effectiveness trials are necessary before taking the intervention into the routine PHC setting for wider community use.

## Supplementary Information

Supplementary material 1: Annex-I. Psychosocial intervention Fidelity Scale.

## Data Availability

The data used in the current study are not publicly available to protect participant confidentiality but are available from the corresponding author on reasonable request.

## Abbreviations

BD: Bipolar Disorder
DALYs: Disability-adjusted life years
LMICs: Low- and middle-income countries
mhGAP-IG: Mental health Gap Action Programme intervention guide
MRC: Medical Research Council’s
PHC: Primary health care
PHCWs: Primary health care workers
PHQ-9: Patient Health Questionnaire-9
PSI-BE: Psychological Intervention for people with bipolar disorder in rural Ethiopia
M(SD): Mean (standard deviation)
SNNPR: Southern Nations, Nationalities, and People’s region
ToC: Theory of change
YMRS: Young Mania Rating Scale
