# Supplementary material for: Feasibility and acceptability of a contextualized brief psychological intervention for people with bipolar disorder in rural Ethiopia
Source: Pilot Feasibility Stud. 2025 Jul 17;11:99. doi: 10.1186/s40814-025-01683-9 (PMC12273223; doi:10.1186/s40814-025-01683-9)
Supplement: Supplementary file 1 — Supplementary material 1: Annex-I. Psychosocial intervention Fidelity Scale. [file 40814_2025_1683_MOESM1_ESM.docx]

**Annex-I: Psychosocial intervention Fidelity Scale**

Date__________________________

Name of assessor = ____________________

Signature: _______________________

Intervention providers name___________

Recording filename __________

Instructions:

Below is an overview of all activities included in the PSI-BE manual session. Please indicate for each activity:

1. Rate how skillfully the contents of the session delivered (competence).
2. How long the session took in practice in the recorded intervention session you rated;
3. whether session content ‘fully completed= 5 (the content was covered in full, as specified in the manual); ‘mostly completed =4 when most aspects of the activity were completed; ‘partly completed=3’ when only a part (50-60% covered) of the activity was completed); ‘some completed=2’ when less than 50% of the contents are covered; ‘not completed=1’ when the activity or some components was skipped entirely.

**Please record each rating next to the item. Overall Session Quality is calculated as an average of all other 1-5 rating scores.**

**Questions used to assess competency the intervention providers in each session**

| No. | Items | Assessor-1 | Assessor-2 | Discussed and agreed score |
| --- | --- | --- | --- | --- |
| 1 | use of appropriate language |  |  |  |
| 2 | Start the session with a general open-ended question |  |  |  |
| 3 | Being sensitivity to participants’ explanations |  |  |  |
| 4 | Uses time efficiently to meet session goals |  |  |  |

**Adherence to the intervention manual (contents described in each session)**

| No. | Items | Assessor-1 | Assessor-2 | Discussed and agreed score |
| --- | --- | --- | --- | --- |
| **Session one: Need assessment and Goal setting** | |  |  |  |
| 1 | Aim of the session |  |  |  |
| 2 | Need assessment |  |  |  |
|  | - Awareness about BD |  |  |  |
|  | - Treatment for BD |  |  |  |
|  | - Awareness about strategies used for promoting wellness |  |  |  |
|  | - Anxiety management |  |  |  |
| 3 | Awareness about action plan |  |  |  |
| 4 | Set the goal in discussion with the participants |  |  |  |
| **Session two: Awareness about Bipolar Disorder** | |  |  |  |
| 1 | Aim of the session |  |  |  |
| 2 | Discussion about BD and its sign and symptoms |  |  |  |
| 3 | Discussion about the sign and symptoms of BD |  |  |  |
| 4 | Discussion on how to recognize early sign and symptoms |  |  |  |
| 5 | Discussion about how to identify influencing factors |  |  |  |
| **Session three: Treatment** | |  |  |  |
| 1 | Aim of the session |  |  |  |
| 2 | Discussion about treatment of the bipolar disorder |  |  |  |
| 3 | Discussion about the side-effect of medication |  |  |  |
| 4 | Discussion about treatment adherence and how to improve treatment adherence |  |  |  |
| **Session four: Promoting Wellbeing of People with BD** | |  |  |  |
| 1 | Aim of the session |  |  |  |
| 2 | Discussed sleep problems and sleep hygiene techniques |  |  |  |
| 3 | Define about stressful situations and their impact on illness |  |  |  |
| 4 | Discussion about interpersonal problems and PST |  |  |  |
| **Session 5:** **Anxiety management and substance use prevention** | |  |  |  |
| 1 | Aim of the session |  |  |  |
| 2 | Discuss on coping mechanisms/anxiety management |  |  |  |
|  | Discussion about breathing exercise and muscle relaxation techniques and their importance |  |  |  |
| 3 | - Breathing exercise practice |  |  |  |
| 4 | - Muscle relaxation techniques practice |  |  |  |
| 5 | Discussed the relapse prevention action plan & its components |  |  |  |
